# Outcomes and process evaluation of a cluster-randomised participatory organisational intervention among German healthcare workers

**DOI:** 10.1186/s12913-023-09240-x

**Published:** 2023-03-16

**Authors:** Diego Montano, Marco Kuchenbaur, Richard Peter

**Affiliations:** 1 Department of Population-Based Medicine, University of Tübingen, Hoppe-Seyler-Str. 9, Tübingen, 72076 Germany; 2 Department of Medical Sociology, Institute of the History, Philosophy and Ethics of Medicine, University of Ulm, Parktstr. 11, Ulm, 89073 Germany

**Keywords:** Effort-reward-imbalance model, Working conditions, Work ability, Occupational health, Participatory interventions

## Abstract

**Background:**

In the present investigation the results of the outcome and process evaluation of a participatory workplace intervention are reported. The intervention aimed to increase the workers’ self-assessed physical and mental work ability.

**Methods:**

The intervention was a two-arm, cluster-randomised trial with healthcare workers in 10 hospitals and one elderly care centre in Germany. Outcome data on workers were collected in questionnaires at baseline, and two follow-ups between 2019 and 2021. The intervention consisted of interviews and workshops, in which employees proposed measures for reducing the physical and psychosocial load and strengthening resources at work. Outcome data were analysed with linear-mixed regression models. The process evaluation was based on the thematic criteria proposed in previous literature and the collection of the type of intervention measures and their implementation status.

**Results:**

The regression analysis did not provide evidence of treatment differences or reductions of psychosocial load in the intervention wards. The process evaluation suggested that the measures did not address specifically the self-assessed work ability. In addition, there was no indication that the intervention measures were causally related to the intended goals.

**Conclusions:**

The planning and implementation of organisational interventions require a careful consideration of the definition of intervention goals, the theoretical rationale of the intervention and a project-oriented action plan during the delivery phase.

## Introduction

Organisational workplace interventions in occupational health can be defined as changes in the working conditions, work processes, organisational policies and procedures, work tasks or the work environment in order to reduce health hazards or improve workers’ well-being [1, 2]. In contrast, individual-oriented interventions aim to change individual behaviour, attitudes, skills or involve the use of personal equipment [3]. Participatory interventions are generally based on the idea that participants of the intervention should contribute their expertise to the determination of the intervention contents, design and implementation [4, 5]. Therefore, it is assumed that participatory organisational interventions are more appropriate for reducing occupational risks at the source and integrating the specific intervention contents into the work routines of the organisation [6].

However, organisational-level workplace interventions are complex social interventions, i.e., they involve several interacting components at both the individual and the aggregate social level which affect the intervention delivery and contents in an unpredictable manner [7]. The challenges associated with complex social interventions are not simply related to whether a particular set of intervention activities are effective or not, but to the circumstances under which those activities are planned, carried out and received by the target groups. Therefore, organisational interventions require not only an evidence-based approach which ensures that the planned measures are effective and the attainment of goals for the targeted groups are feasible [2, 8], but also an appropriate strategy which serves as a guide for the actual implementation of the intervention [9, 10]. At the same time, the complexity inherent to organisational interventions demands an evaluation of the expected outcomes in terms of effect size estimates and the intervention process itself as well. Whereas the outcome evaluation is usually performed by statistical analysis suitable for a specific study design, the evaluation of the intervention process is much more heterogeneous. Several process variables have been used in previous research to assess the implementation quality of interventions in occupational health including contextual factors, barriers and facilitators, initiation of the intervention, ownership, appropriateness, participation, protocol adherence, communication, management support and readiness for change, among many others [11, 12].

Health services providers in several European countries are currently confronted with some form of personnel shortage due to several factors including an ageing workforce and the relatively high levels of psychosocial risk and ensuing mental health symptoms including long working hours, job insecurity, burnout and stress in the health care sector [13]. Work in healthcare is concomitant with increased emotional demands [14], high cognitive and time demands [15] and low job rewards [16]. As a response to this situation in the health-care labour market, some health services providers have been implementing in recent years some form of age management practices to reduce the impact of this personnel shortage [17]. In a previous study with health care organisations in Germany, the UK and Finland, it was found that the most frequent age management measures concerned reductions of the working time or re-arrangement of work scheduling [17]. Nonetheless, the findings suggested that health care organisations do not usually attempt to decrease work demands, modify the work environment or adopt a life-course approach with special emphasis on age and career phases or healthcare workers [17].

From a more general perspective, these type of age management programmes can be interpreted as occupational health interventions focusing on working time arrangements and shifting schedules of healthcare workers. However, there are several research gaps regarding the expected primary outcomes resulting from such programmes. For instance, the mechanisms are not specified whereby the rescheduling of working time arrangements should reduce personnel shortages in healthcare settings. Even though the redesign of shift work schedules may have some beneficial effects on outcomes such as work-life balance and work stress [18, 19], it is unclear whether previous age management programmes actually targeted such outcomes as antecedents of staff turnover or early retirement intentions. In addition, to the knowledge of the authors, previous age management programmes in healthcare settings have not been evaluated in randomised controlled trials and, therefore, there is a high risk of bias in the corresponding literature.

Hence, the present study contributes to previous research in occupational health interventions in two ways: First, the study presents the results of a participatory organisational intervention which explicitly addressed the age and career phases of healthcare workers to improve their perceived work ability and, hence, increase the chances of longer employment careers of workers. Second, the present study reports not only the effect size estimates of the intervention, it also focuses on the psychosocial load in terms of the Effort-Reward Imbalance (ERI) model and provides a detailed process evaluation which addressed the context and actual delivery of the intervention. The ERI model of work stress assumes that the perception of lack of reciprocity in terms of high efforts and low rewards at work elicit stress reactions [20]. The ERI model postulates also that failure to withdraw from work obligations, i.e., to be overcommitted to one’s own work duties, represents a health-adverse coping pattern [20]. Hence, the ERI model is based on three dimensions, namely, efforts, low rewards and overcommitment. Against this background the present study provides additional information which may help to understand the impact of the intervention on work stress and how and why the observed outcomes may have come about [2, 8, 10]. In addition, considering that the present intervention started before the declaration of the COVID-19 pandemic, it was possible to assess the potential impact of the pandemic on the primary and secondary intervention outcomes. This is important given the fact that healthcare workers may have been more exposed to a stressful work environment during the early stages of the Sars-CoV-2 outbreak [21].

## Methods

### Study design and data

The intervention “HALTgeben” (“Higher Patient Satisfaction through Fair Working Conditions in Healthcare”) was conducted among healthcare workers in 11 German health services institutions, namely, seven general and three specialised hospitals and one elderly care centre. The intervention was motivated by the personnel management of these health services institutions following the invitation to participate in the study. These institutions were selected because they are located near the consultants’ offices and have a sufficient number of workers and patients required to assess the effects of the intervention on the main intervention outcomes. The main aim of the intervention was to improve the self-assessed physical and mental work ability of healthcare workers by modifying the working conditions in the intervention wards. The intervention was conducted in a two-arm, cluster-randomised design whose study protocol and baseline results have already been published elsewhere [22]. Eligible participants were healthcare workers 18 years and older who worked most of the time in a single ward. All eligible healthcare workers were invited to participate in the study. The clusters were built by aggregating wards of similar medical disciplines (e.g., anaesthesiology, intensive care units, neurology, etc.) and located in separate building areas. Only wards in which at least one healthcare worker consented to participate in the study were included. The randomisation of clusters was performed with assignment probabilities proportional to size. A total of 10 cluster were allocated to the intervention arm. Results at baseline suggested that the random allocation of clusters was satisfactory, for there were no differences between the intervention and control arms regarding the primary and secondary outcomes [22]. The power analysis conducted at baseline showed that effect sizes of at least 0.30 and 0.27 for physical and mental work ability can be estimated at the 80% power and 5% significance levels. Given the effect size estimates reported in previous workplace interventions, the present study was found to have sufficient power to detect substantial changes in the main outcomes [22]. Primary outcomes were the self-assessed physical and mental work ability of healthcare workers who participated in the surveys.

The survey data used in the present study was collected in the intervention and control arms at baseline (T0) and two follow-up times (T1 and T2) between June to December 2019, September to December 2020 and August to October 2021, respectively. Healthcare workers who gave informed consent to participate in the surveys were able to fill the questionnaires either online or on paper. All workers in the intervention wards were given the chance to participate in the workshops delivered in the intervention (see below). However, not all workers in the single intervention wards took part in the surveys. Outcomes were measured by appropriate validated psychometric instruments comprising physical and psychosocial working conditions, work ability, and perceived physical and mental health (see [22] for more details on the scales). For the purposes of the present study the following data were considered:

1. age and sex.
2. The physical and mental work ability as measured with two items of the Work Ability Index Questionnaire “How would you appraise your current work ability in relation to the physical work demands?” and “How would you appraise your current work ability in relation to the mental work demands?”, respectively, with answer format ranging from 1 to 5 as follows: 1: poor, 2: not good, 3. good, 4: very good and 5: excellent [23].
3. The scales of the ERI Questionnaire effort (6 items), reward (11 items) and overcommitment (6 items), with answer format: 1: strongly disagree, 2: disagree, 3: agree and 4: strongly agree [24].
4. In addition, information on ward transfers was collected at the T1 and T2 follow-ups since these could have resulted in confounding effects, especially due to the disruptions in the healthcare system during the first year of the COVID-19 pandemic in 2020.

### Statistical analysis

**Fig. 1:**
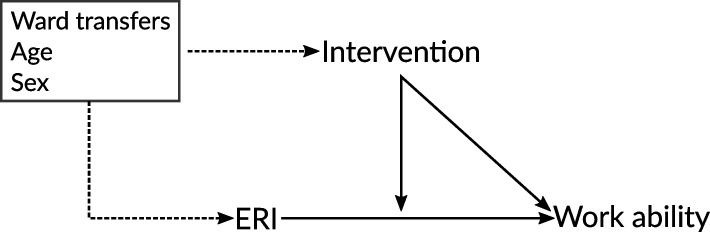
Graph of the assumed causal process in the statistical models. ERI: Effort-Reward Imbalance scales. Dotted line indicates potential confounding effects

The intervention effects on the primary outcomes were estimated by means of generalised linear mixed-effects regression models [25]. Treatment differences between the two study arms were expected to be positive, i.e., it was hypothesised that the average self-assessed work ability of healthcare workers in the intervention arm would be higher than in the control arm. The regression estimates were calculated with all data collected during the study period in order to adjust for potential confounding related to sample attrition (intention-to-treat approach) [26]. In addition, given that the intervention addressed the psychosocial load at work, the scales of the ERI questionnaire entered the analysis as moderating variables in order to assess the impact of the intervention on the psychosocial load in the intervention wards. The main hypothesis of the intervention study is assessed in model 1 by estimating the treatment effects. In model 2, the regression model 1 was expanded by including the three scales of the ERI questionnaire, namely, efforts, low rewards and overcommitment. Finally, the main and interaction effects of treatment assignment and the ERI scales are estimated in model 3. Even though the randomisation ensured the comparability of both intervention arms, all models take into consideration the potential confounding effects of ward transfers and age and sex as additional explanatory variables in order to explore systematic differences over time which may be attributable to increasing age or sex [27]. The confounding effects were assumed to affect primarily the intervention itself and the ERI dimensions. The regression models depicted in Fig. 1 were estimated in an intention-to-treat approach as stated above.

### The intervention

The intervention was based on the concept of work ability which refers, in general, to the combination of the information about the workers’ health status and their appraisal of their own ability to meet the job demands [28, 29]. The intervention was conducted by consultants working in the field of work design and organisational development. The intervention was conceived to help workers accomplish their work duties by considering how their individual characteristics and capacities may be aligned with the specific work and task processes. It was expected that this alignment would ultimately foster the perception of an increased work ability. The consultants’ approach specified four age-dependent main career stages: entrance, development and transition, continuity and exit [30].

The intervention consisted of four phases:

1. *Phase*. The consultants assessed the organisational structure by collecting different sources of information such as work tasks descriptions of targeted groups, shift schedules or risk assessments.
2. *Phase*. The consultants conducted semi-structured interviews with voluntary employees and supervisors in the intervention wards to discuss about aspects of the work organisation and process, workload and psychosocial demands.
3. *Phase*. The consultants organised and moderated workshops lasting about 3 h in which the participating healthcare workers and supervisors were encouraged to propose measures to enhance the workers’ work ability, improve the working conditions in the wards and adapt the work environment to an ageing workforce. The results of the semi-structured interviews were summarised by the consultants according to the main themes described by the interviewees and the main components of the work-ability model. For instance, the perception of interviewees concerning work tasks, duties, work processes, conflicts, individual capacities, leadership, motivation, career stages, health issues and potential solutions to the most pressing problems in the wards were presented at the beginning of the workshop. Workshop participants were then asked to discuss the issues mentioned in the interviews and to give their own view on these and other work-related matters which they considered important for their work ability and career stages (i.e., entrance, development and transition, continuity and exit).
4. *Phase*. The so-called “initiatives circles” were established which consisted of managing board executives, managers of the healthcare departments and representatives of the works council, quality management or human resources department. The initiatives circles had the responsibility to appraise the feasibility of the intervention measures, and to accept or reject the measures proposed in the workshops, with the exception of measures which were affected by legal constraints (e.g., labour agreement stipulations). The initiatives circles decided which measures could be implemented by the wards themselves and which required further executive board approval.

The intervention approach assumes that the proposed measures made by the workshop participants would result in improvements of the working conditions in the particular wards. Against this background, it was expected that all healthcare workers in the intervention wards, even workers who did not take part of the workshops or interviews, would benefit from the implemented measures and, therefore, they would experience an increase of their self-assessed work ability.

### Process evaluation

The process evaluation was performed by the authors at the University of Ulm, independently from the consultants conducting the intervention. The appraisal of the implementation process followed the thematic list proposed by Egan and colleagues which includes the following criteria [10].

1. *Motivation*. Who initiated the intervention?
2. *Organisational change*. Was the intervention based on a theory of change?
3. *Context*. In which context was the intervention embedded (e.g., political, managerial)?
4. *Experience*. Did the individuals responsible for implementing the intervention have experience with organisational change or, if not, did they receive appropriate training?
5. *Consultations*. Did the intervention include planning consultations?
6. *Delivery*. Were there delivery collaborations among the participants?
7. *Manager support*. Were managers supportive of the intervention?
8. *Employee support*. Were employees supportive of the intervention?
9. *Resources*. Which resources are required in implementing the intervention?
10. *Differential effects*. Did the intervention have differential effects, e.g., some people benefiting more from the intervention, harmful effects, etc.?

The contents of the intervention measures proposed in the workshops were recorded by the consultants in a spreadsheet and forwarded to the authors located at the University of Ulm who classified the proposed measures according to the categories developed by Giga et al. (2003) [31]. In order to ease the presentation of results, the measures are reported according to the following categories:

1. Individual-level intervention measures including participation and autonomy measures, person-environment-fit, reward schemes, role issues, employee assistant programmes, exercise and relaxation programmes.
2. Organisational-level measures divided into:
  - physical and environmental characteristics (PEC);
  - selection and placement (SAP) policies;
  - training and education (TRA) programmes;
  - work processes and working time (WPT), and
  - other measures at the organisational level.

The classification was performed independently by each one of the authors of the present study and discrepancies were solved by discussion. Measures that could not be classified in one of the categories of Giga et al. were labelled “unclassifiable”. The status of the actual implementation of the proposed measures by end June 2021 was reported by the consultants to the University of Ulm in three categories: implemented, not implemented and unknown. The impact of the intervention as perceived by the healthcare workers who participated in the surveys was measured by the single item: “To what extent has the research project HALTgeben brought about changes of your work situation?”, with answer categories: improvement, not change at all, not aware of the intervention and worsening of the work situation.

## Results

### Statistical analysis

The descriptive statistics of the sample and the correlation matrix are provided in Table 1. Concerning the primary outcomes of the intervention, the results of the regression analysis did not provide support to the hypothesis that the intervention would improve the self-assessed work ability among healthcare workers in the intervention arm in comparison to the control arm. The estimated treatment differences obtained from model 1 did not reveal substantial differences between the self-assessed physical and mental work ability in the study arms (-0.07, 95% CI [-0.24; 0.09] and -0.05, 95% CI [-0.22; 0.11], respectively). In fact, workers in the intervention arm tended to report lower physical and mental work ability (models 1 and 2 in Table 2). On the other hand, the results obtained in models 2 and 3 revealed large associations between the ERI scales efforts, low rewards and overcommitment and both physical and mental work ability. Whereas the efforts scale seemed to be more related to physical rather than mental work ability, the opposite was the case for the overcommitment scale whose effect size estimates were larger for mental rather than physical work ability (Table 2). Furthermore, the interaction effects model suggested that the intervention did not have any influence on the psychosocial load at work as measured by the ERI scales, given the fact that none of the interaction terms indicated large treatment differences (Table 2).

**Table 1:** Correlations at baseline, proportions (%) of selected variables and sample sizes of the intervention ($N_i$) and control arm ($N_c$). Cronbach’s $\alpha$ of the scales (bold) on the diagonal of the correlation matrix. NA: Cronbach’s alpha not defined for the single items WAI-P and WAI-M

	WAI-P	WAI-M	Efforts	Low rewards	Overcommitment
WAI-P	NA				
WAI-M	0.45	NA			
Efforts	-0.44	-0.38	**0.81**		
Low rewards	-0.29	-0.32	0.49	**0.81**	
Overcommitment	-0.31	-0.44	0.57	0.41	**0.80**
Age (%)	18-39y: 23; 40-54y: 48, 55y and older: 29				
Sex (%)	Male: 21, Female: 79				
$N_i$	$t_0$: 240, $t_1$: 186, $t_2$: 167				
$N_c$	$t_0$: 146, $t_1$: 116, $t_2$: 103				
Attrition	from $t_0$ to $t_1$: 22%, from $t_0$ to $t_2$: 30%				

WAI-P: physical work ability, WAI-M: mental work ability, NA: not available

**Fig. 2:**
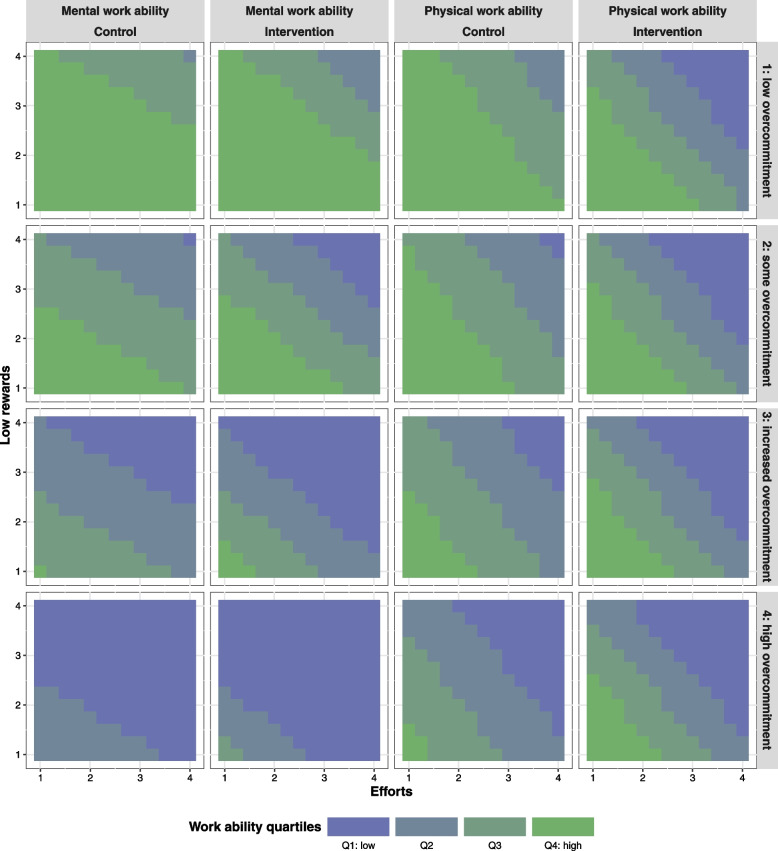
Estimated marginal effects of efforts, low rewards and overcommitment on physical and mental ability by treatment. Estimates obtained from regression model 3

In order to ease the interpretation of the results obtained in the regression analysis, in particular for the interaction model, the marginal effects of efforts and low rewards on physical and mental work ability by levels of overcommitment are depicted in Fig. 2. It can be observed that the combination of high efforts and low rewards is associated with lower work ability levels, especially as the intensity of perceived overcommitment increases. For instance, given a low level of overcommitment (top panel row in Fig. 2), the level of work ability is high, especially for mental work ability and among workers in the control wards. On the contrary, given the highest level of overcommitment (bottom panel row in Fig. 2), the work ability is lowest, even if the job efforts are low and rewards high, i.e., even if the psychosocial workload remains at low levels.

Finally, the regression models revealed that healthcare workers who did not experience ward transfers reported higher work ability levels than transferred workers. However, the analyses did not suggest that the COVID-19 disruptions had a larger impact on work ability. In fact, there were only about 13% ward transfers during the intervention period, with COVID-19 related transfers or changes accounting for only 30% of ward transfers. In total, only about 7% of the survey participants were affected by some COVID-19 related changes during the intervention. Hence, the results of the regression models concerning ward transfers indicate that transfers in themselves posed increased demands on transferred workers and, thus, may have contributed to lower work ability, especially for its mental component. On the other hand, while the analysis indicated that physical, but not mental work ability, decreases with increasing age, the role of sex was less consistent and seemed to be fully mediated by the psychosocial workload. This can observed by taking into consideration that sex did not contribute to the proportion of explained variance of the work ability components in models 2 and 3, in which the ERI scales are taken into account. Hence, the observed sex-specific differences regarding work ability are likely due to differences in the perception of the psychosocial workload as measured by the ERI scales.

**Table 2:** Generalised linear regression models. Dependent variables: physical and mental work ability, WAI-P and WAI-M, respectively. Regression coefficients and 95% confidence intervals in brackets. $N_{obs}$: number of observations, $N_{ind}$: number of individuals

	Model 1		Model 2		Model 3	
Variable	WAI-P	WAI-M	WAI-P	WAI-M	WAI-P	WAI-M
Intercept	3.53 [3.20; 3.86]	2.97 [2.64; 3.30]	5.02 [4.61; 5.42]	4.86 [4.46; 5.26]	4.98 [4.53; 5.43]	4.67 [4.23; 5.12]
Age	-0.07 [-0.11; -0.04]	-0.01 [-0.05; 0.02]	-0.07 [-0.11; -0.04]	-0.01 [-0.05; 0.02]	-0.07 [-0.11; -0.04]	-0.01 [-0.05; 0.02]
Female (Ref. Male)	-0.22 [-0.43; -0.02]	-0.25 [-0.46; -0.05]	-0.07 [-0.25; 0.12]	-0.05 [-0.23; 0.14]	-0.06 [-0.24; 0.13]	-0.05 [-0.23; 0.13]
Intervention (Ref. control)	-0.07 [-0.24; 0.09]	-0.05 [-0.22; 0.11]	-0.08 [-0.23; 0.06]	-0.08 [-0.22; 0.07]	0.05 [-0.60; 0.70]	0.53 [-0.11; 1.17]
Intervention x efforts					-0.11 [-0.35; 0.13]	-0.13 [-0.36; 0.11]
Intervention x low rewards					-0.12 [-0.37; 0.14]	-0.08 [-0.33; 0.17]
Intervention x overcommitment					0.14 [-0.11; 0.38]	-0.06 [-0.30; 0.18]
Efforts			-0.30 [-0.41; -0.18]	-0.19 [-0.31; -0.08]	-0.26 [-0.40; -0.11]	-0.14 [-0.29; -0.00]
Low rewards			-0.25 [-0.37; -0.12]	-0.27 [-0.39; -0.15]	-0.20 [-0.36; -0.04]	-0.25 [-0.40; -0.09]
Overcommitment			-0.16 [-0.28; -0.04]	-0.41 [-0.53; -0.30]	-0.22 [-0.36; -0.07]	-0.40 [-0.54; -0.25]
Not transferred (Ref. transferred)	0.21 [0.03; 0.39]	0.35 [0.17; 0.54]	0.20 [0.02; 0.38]	0.35 [0.17; 0.52]	0.20 [0.02; 0.37]	0.34 [0.17; 0.52]
No information on ward transfer	-0.06 [-0.37; 0.25]	0.13 [-0.18; 0.45]	-0.01 [-0.33; 0.30]	0.17 [-0.14; 0.48]	-0.02 [-0.33; 0.30]	0.17 [-0.14; 0.48]
$N_{obs}$	916	915	869	870	869	870
$N_{ind}$	385	385	377	378	377	378

### Process evaluation

The process evaluation revealed that the majority of proposed interventions addressed the physical and environmental characteristics of the workplace, namely, about 17% of the 524 measures recorded by the consultants. In contrast, individual measures accounted for just about 6% of all proposed measures. By the end of the intervention, only 22% of the measures were actually accomplished. However, the largest proportion of measures (about 46%) could not be appropriately classified due to the fact that, in most instances, it was not feasible to assess which work component was addressed by the measures. For example, there was a large number of “measures” recorded by the consultants which were actually either discussions on polemic topics, complaints about certain work situations or measures without a specific goal (see some examples of the type of proposed measures in Table 3). From the perspective of the healthcare workers who participated in the surveys, about 69% thought that the intervention did not brought about any changes to their working conditions (Table 4). Less than five workers thought that the intervention worsened their working conditions, whereas about a third was not even aware of the intervention. In general, there were practically no differences in the perceived impact of the intervention between the intervention and control arms. Beside some delays in the scheduling of appointments and the delivery of measures, there was no indication in the process evaluation that the COVID-19 pandemic had a large impact on the intervention and its outcomes.

**Table 3:** Examples of two proposed measures by intervention level

ID	Measure	Level
43	Physiotherapy for healthcare workers should be offered	1-Individual
91	Healthcare workers should be relieved from tasks not related to patients’ care	1-Individual
107	Special medical beds are needed for patients requiring intensive care	2-PEC
286	A lift for the laboratory is needed; in general, the walk distances should be reduced	2-PEC
76	Patient transport staff is needed for the night shift	3-SAP
314	The composition of work teams should be considered when scheduling work	3-SAP
143	Kinaesthetics training should be offered and discussed in human resources development talks	4-TRA
49	Electrocardiogram advanced training for the whole ward should be offered	4-TRA
19	The posture of healthcare workers should be improved when instructing mothers on breast feeding and conducting audiometry tests on newborns	5-WPT
33	Fixed time slots should be defined for talking to patients and relatives in order to avoid interruptions of one’s work duties	5-WPT
145	Physicians should give feedback during team meetings	6-Other organisational
313	The discussion of patient cases should be better organised, i.e., the meetings should begin and end on time, the discussion should be structured and moderated and some solutions or decisions need to be summarised	6-Other organisational
104	Workshop participants discussed about the general mission of the hospital	7-Unclassifiable
21	The offer of swimming facilities should be mentioned for new colleagues who are not familiar with it	7-Unclassifiable

PEC: Physical and environmental characteristics, SAP: Selection and placement, TRA: Training and education programmes, WPT: Work processes and working time

In terms of the appraisal of the implementation process regarding the thematic list proposed by Egan and colleagues, the process evaluation yielded following results:

1. *Motivation*. The intervention was motivated by the management of the participating health services providers.
2. *Organisational change*. The intervention lacked of a theory of organisational change and there were no strategies to follow-up and guarantee the actual delivery of measures.
3. *Context*. The implementation was performed in the context of the ageing workforce in the healthcare sector in Germany and the personnel strategy of the management of the participating providers.
4. *Experience*. There was no training for the individuals who were responsible for implementing the intervention, i.e., the workers, supervisors, line managers, and the personnel of the human resources and quality management. Most intervention measures were actually not implemented due to reasons including the rejection of some measures in the initiatives circles, the lack of clear responsibilities regarding the implementation, unclear goals of the measure or inappropriateness of the measure itself.
5. *Consultations*. There were planning consultations between managers and healthcare workers since the intervention was based on a participatory approach in which the measures were proposed by the workers themselves.
6. *Delivery*. In the course of the intervention, there were no delivery collaborations between managers, workers or any other relevant parties to monitor and ensure the progress of the implementation of the measures.
7. *Manager support*. Healthcare workers participating in the workshops believed that their mangers would support the intervention.
8. *Employee support*. Healthcare workers participating in the workshops had a relatively high expectation that the measures could be implemented in the wards.
9. *Resources*. The intervention approach did not consider the planning and allocation of resources. There was no guidance to assess the budgetary implications of measures.
10. *Differential effects*. Although there were differential effects of the intervention (Table 4), the workers’ perception of the intervention was that it did not bring about any changes at all in the working conditions.

**Table 4:** Healthcare workers’ appraisal of the intervention effects on their own working conditions. Frequencies and proportions in parentheses

Appraisal	Control	Intervention	Total
Improvement	6 (4%)	9 (9%)	15 (6%)
No change at all	106 (67%)	74 (74%)	180 (69%)
Not aware of the intervention	46 (29%)	15 (15%)	61 (24%)
Worsening	less than 5 (–)	less than 5 (–)	less than 5 (–)
**Total**	159 (100%)	100 (100%)	259 (100%)

## Discussion

The statistical analysis did not provide support to the hypothesis that the intervention would improve the self-assessed physical and mental work ability of healthcare workers in the intervention wards. There was also no evidence that the intervention was beneficial for reducing the psychosocial load at work in terms of the efforts, rewards and overcommitment perceived by the workers receiving the intervention. Furthermore, the results obtained from the process evaluation did not suggest that the implemented measures were specifically addressing the self-assessed physical and mental work ability. Since the intervention lacked an evidence-based approach to organisational change, the actual delivery of the measures was not embedded in a systematic plan to monitor and ensure the implementation of measures. For the majority of healthcare workers (about 70%), the intervention study did not have any impact whatsoever on their working conditions. This finding indicates that the main pillar of the intervention, i.e., the organisation and moderation of single workshops with selected workers and supervisors in the wards, was not conducive to the pursued improvements of the self-assessed physical and mental work ability. Even though the disruptions related to COVID-19 resulted in few workers being transferred to the intensive care units, the findings suggested that that ward transfers as such had a negative impact on the self-assessed work ability, independently of the cause behind the transfer (Table 2). Although few wards reported that the COVID-19 related disruptions hindered or delayed the implementation of some measures, these was no indication that these disruptions prevented the delivery of the proposed measures in all wards and institutions.

The failure of the intervention to attain the proposed goals can be explained by taking into account some theoretical considerations pertaining to the conduction of complex social interventions. It seems that the main deficiency of the present intervention was the lack of an evidence-based set of statements providing the rationale of why certain intervention measures may be causally related to the expected outcomes. The intervention was rather vague concerning the specific causal mechanisms which were thought to lead to the expected outcomes. The recurrence to the work ability concept and the definition of specific primary endpoints were indeed explicit, but there was no decision guide for the relevant actors (workers, supervisors, management) as to the type of measures which would result in improvements of the self-assessed work ability of healthcare workers. In particular, the analysis of the single intervention measures revealed that the healthcare workers participating in the workshops did not have a clear idea of what an intervention implies, namely, to change some aspects of the working environment [32, 33]. Most “measures” could not be related to specific changes of the working conditions, but were rather complaints, individual requests, anecdotes or issues being debated during the workshops (about 46% of all “measures”, Table 5). Even though one of the strengths of the intervention was the participatory approach, there was not systematic approach of how to select the most effective measures leading to improvements of the self-assessed work ability. As a matter of fact, the analysis suggested that some wards may have even experienced a worsening of the working conditions (Table 4).

**Table 5:** Intervention level of measures and their implementation status in the intervention wards

Intervention level	Implementation status			Total
	Accomplished	Not accomplished	Unknown	
1-Individual	8 (26%)	23 (74%)	0 (0%)	31 (6%)
2-PEC	17 (19%)	67 (76%)	4 (5%)	88 (17%)
3-SAP	4 (14%)	19 (66%)	6 (21%)	29 (6%)
4-TRA	7 (14%)	40 (82%)	2 (4%)	49 (9%)
5-WPT	11 (19%)	41 (72%)	5 (9%)	57 (11%)
6-Other organisational	12 (43%)	15 (54%)	1 (4%)	28 (5%)
7-Unclassifiable	59 (24%)	175 (72%)	10 (4%)	244 (46%)
**Total**	118 (22%)	380 (72%)	28 (5%)	526 (100%)

PEC: Physical and environmental characteristics, SAP: Selection and placement, TRA: Training and education programmes, WPT: Work processes and working time

Moreover, despite the fact that the contents discussed during the workshops did focus on key issues including the adequacy of work processes, extent of job duties, leadership or health issues of healthcare workers in the intervention wards, the final decision on the implementation of measures was not always made by the workers themselves. In particular, measures which affected more structural aspects of the work environment, e.g., definition of work tasks, work load, coordination of work within and between wards, were competency of the initiative circles. From this perspective, the intervention approach did not explicitly defined feedback or consultation mechanisms between the wards and the initiative circles. Hence, it seems that the key limitation concerning the efficacy of the workshops as the centrepiece of the intervention was not primarily due to the specific contents and themes discussed in the intervention, but rather to different factors associated with the identification and selection of effective measures, the lack of a theoretical rationale for defining and prioritising the measures and the partial detachment of decision-making power from the intervention wards.

It has to be acknowledged that the receptivity and engagement of the healthcare workers themselves [34], as measured by the concepts of workshop-related efficacy expectation and prospective outcome expectations, was rather high, as additional analyses focusing on the collective self-efficacy beliefs of the workshop participants indicated [35]. There was some evidence suggesting that the higher the workshop-related efficacy expectation, the larger the number of proposed measures was. Hence, it seems that the intervention activities were strongly supported by workers and supervisors. Since collective self-efficacy refers to people’s shared beliefs in their collective power to produce desired results by collective action [36], there seemed to have been sufficient receptivity and engagement among workers to bring about changes in the working conditions at the ward level. However, as stated above, the participation of workers was not embedded in a general framework of causal mechanisms relating the proposed measures and the intended goals and, therefore, there was indeterminacy regarding the results to be expected from the collective action efforts. In addition, the intervention approach consisted of a single workshop and, therefore, it did not provide continuous support throughout the intervention period to enable workers revise the adequacy of measures and ensure their delivery in the intervention wards.

The analysis of the associations between the psychosocial workload and the physical and mental work ability did not provide evidence that the intervention resulted in a reduction of job efforts and overcommitment, or in the improvement of the rewards obtained at work in the intervention wards. Nonetheless, these results emphasised once more that these psychosocial risk factors had a large impact on the perceived work ability and, consequently, confirmed previous research findings obtained with larger samples of the employed population in Germany and Finland which reported substantial associations between the effort-reward-imbalance ratio and the general work ability [37, 38]. Moreover, the observation that high efforts and low rewards are related to an increased likelihood of sick leave [39] and the intention to claim disability pension [37] underlines the importance of maintaining lower levels of psychosocial workload. At the same time, these findings point to potential causal mechanisms which may inform the design of future organisational interventions which explicitly address the work characteristics specifically associated with job efforts, rewards and overcommitment. On the basis of previous interventions it can be expected that such an evidence-based approach may be more effective in attaining beneficial outcomes at the individual and organisational level [40, 41].

### Implications

By taking into consideration the results of the present participatory organisational intervention, it can be concluded that at least three major criteria may help organisations and researchers with the design and planning of more effective participatory organisational interventions.

1. *Definition of the intervention goals*. The present intervention pursued to improve the self-assessed physical and mental ability. However, the concept of work ability is rather an attitude, i.e., a cognitive appraisal process of one’s prospects of coping with the physical and mental job demands [42]. The intervention approach implicitly assumed that this cognitive appraisal could be changed by delivering the modifications proposed by the healthcare workers. However, previous research in social psychology has indicated that the modification of attitudes is a challenging task which requires strong stimuli and effective environmental modifications [43]. Even though it appeared plausible to assume that the self-assessed work ability could be changed by modifying the working conditions as proposed by the workers, the actual causal pathways of how those proposed measures would serve the intervention goals were not identified. In addition, it was assumed that the measures proposed in the workshops would be beneficial for all workers in the wards, independently of whether they participated in the workshops or not. However, since the workers participating in the survey were in rare instances also participants of the workshops, the lack of treatment effects indicates that this assumption was not tenable, i.e., no spill-over of benefits were observed for all workers in the intervention wards. Accordingly, workplace interventions should carefully take into consideration the feasibility of achieving the goals and, accordingly, identify the set of modifications which may be causally related to the intended goals.
2. *Intervention approach*. The process evaluation indicated that the intervention limited itself to the preparation of some activities including the establishment of initiatives circles, the conduction of interviews and the organisation and moderation of single workshops. However, the intervention approach did not take into consideration that workplace interventions require continuous monitoring, evaluating and adjusting of the intervention processes and contents [5, 9]. As the process evaluation revealed, the delivery of the intervention was unsuccessful since no mechanisms were installed to manage the organisational change activities, i.e., to check and revise contradictory or ineffective measures, bypass unforeseeable events (e.g., COVID-19 pandemic) or facilitate the coordination of the intervention measures within and across wards [8]. Over time, the intervention efforts waned and the majority of proposed measures were not implemented. Consequently, any approach to conduct organisational interventions should be conceived as an ongoing organisational change process based on a feedback system including at the very least the phases of preparation of the intervention, selection of methods, action planning, monitoring of the implementation and evaluation [9].
3. *The action plan*. The intervention did not provide explicit guidance regarding timelines for the implementation of measures or the allocation of resources necessary to deliver the measures. The vagueness of the goals was accompanied thus by the vagueness of action plans to implement the proposed measures. The intervention failed to identify the specific activities which were needed in order to bring about specific organisational changes such as procurement of resources, budgeting, the clear delegation and assignment of tasks and responsibilities or the mechanisms used to maintain and enforce the measures [8]. Form this perspective, workplace interventions would greatly benefit by adopting a project-based approach in which tasks, responsibilities, resource allocation and timelines are specifically determined in order to bring about actual changes in the organisational structure and processes.

### Strengths and limitations

The major strengths of the present intervention are the study design and the extensive process evaluation. In contrast to other organisational interventions which are prone to confounding due to several factors including lack of control groups, randomisation or treatment contamination [1], the results of the present study are robust given the successful implementation of a cluster-randomised controlled design [22]. In addition, the process evaluation was based on previous literature and included group and individual levels of variation [35] which allowed an in-depth analysis of the most important factors which may have contributed to the observed results. On the other hand, there are two major limitations. First, the information on the intervention measures proposed in the workshops was collected by the consultants and not by independent observers. Even though the information on the workshop contents and implementation status was systematically collected, there were ambiguities in the description of single measures. However, since the measures were classified independently by each author of the present study, the impact of those ambiguities can be considered low. Second, due to organisational constraints it was not feasible to collect detailed information of the work in the initiatives circles. Hence, the analysis of the decision-making process leading to the acceptance or rejection of measures could not be performed.

## Data Availability

The datasets generated and analysed during the current study are not available to third parties due to the German data protection regulations under which this intervention was conducted.
